# 2-[3-(1*H*-Benzimidazol-2-yl)prop­yl]-1-decyl-1*H*-benzimidazole

**DOI:** 10.1107/S160053681103501X

**Published:** 2011-08-31

**Authors:** Hamid Ennajih, Rachid Bouhfid, El Mokhtar Essassi, Seik Weng Ng

**Affiliations:** a Moroccan Advanced Science, Innovation and Research Foundation, Institute of Nanomaterials and Nanotechnology, Rabat, Morocco; b Laboratoire de Chimie Organique Hétérocyclique, Faculté des Sciences, Université Mohammed V-Agdal, Rabat, Morocco; c Department of Chemistry, University of Malaya, 50603 Kuala Lumpur, Malaysia; d Chemistry Department, Faculty of Science, King Abdulaziz University, PO Box 80203 Jeddah, Saudi Arabia

## Abstract

The asymmetric unit of the title compound, C_27_H_36_N_4_, contains two independent mol­ecules. Except for the atoms of the decyl chain, the non-H atoms of each mol­ecule are approximately coplanar (r.m.s. deviations = 0.075 and 0.164 Å) and the –CH_2_CH_2_CH_2_– link connecting the two benzimidazolyl fused-ring systems is slightly opened up at the middle C atom. The decyl substituent adopts an extended zigzag conformation in both mol­ecules. In the crystal, adjacent mol­ecules inter­act by N—H⋯N hydrogen bonds, generating a chain parallel to the *c* axis.

## Related literature

For 2,2′-(propane-1,3-di­yl)bis­(benzimidazolium) dichloride and hydrogen perchlorate, see: Hu *et al.* (2006 ▶); Sun *et al.* (2004 ▶).
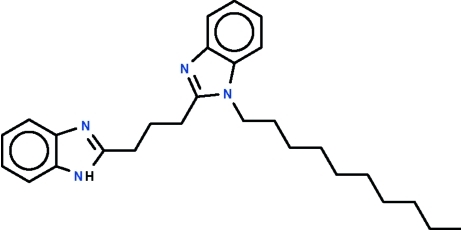


## Experimental

### 

#### Crystal data

C_27_H_36_N_4_
*M* *_r_* = 416.60
Monoclinic, 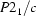

*a* = 38.5580 (6) Å
*b* = 13.2052 (2) Å
*c* = 9.9623 (2) Å
β = 95.339 (1)°
*V* = 5050.46 (15) Å^3^
*Z* = 8
Mo *K*α radiation
μ = 0.07 mm^−1^
*T* = 293 K
0.30 × 0.20 × 0.10 mm

#### Data collection

Bruker APEXII diffractometer
38663 measured reflections
9083 independent reflections
5547 reflections with *I* > 2σ(*I*)
*R*_int_ = 0.038

#### Refinement

*R*[*F* ^2^ > 2σ(*F* ^2^)] = 0.056
*wR*(*F* ^2^) = 0.165
*S* = 1.02
9083 reflections
567 parameters
36 restraints
H atoms treated by a mixture of independent and constrained refinement
Δρ_max_ = 0.27 e Å^−3^
Δρ_min_ = −0.20 e Å^−3^

### 

Data collection: *APEX2* (Bruker, 2005 ▶); cell refinement: *SAINT* (Bruker, 2005 ▶); data reduction: *SAINT*; program(s) used to solve structure: *SHELXS97* (Sheldrick, 2008 ▶); program(s) used to refine structure: *SHELXL97* (Sheldrick, 2008 ▶); molecular graphics: *X-SEED* (Barbour, 2001 ▶); software used to prepare material for publication: *publCIF* (Westrip, 2010 ▶).

## Supplementary Material

Crystal structure: contains datablock(s) global, I. DOI: 10.1107/S160053681103501X/xu5311sup1.cif

Structure factors: contains datablock(s) I. DOI: 10.1107/S160053681103501X/xu5311Isup2.hkl

Supplementary material file. DOI: 10.1107/S160053681103501X/xu5311Isup3.cml

Additional supplementary materials:  crystallographic information; 3D view; checkCIF report

## Figures and Tables

**Table 1:** Hydrogen-bond geometry (Å, °)

*D*—H⋯*A*	*D*—H	H⋯*A*	*D*⋯*A*	*D*—H⋯*A*
N1—H1⋯N2^i^	0.88 (1)	1.96 (1)	2.813 (2)	162 (2)
N5—H5⋯N6^ii^	0.88 (1)	2.01 (1)	2.860 (2)	162 (2)

Symmetry codes: (i) 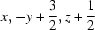; (ii) 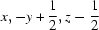.

**Fig. 1.**
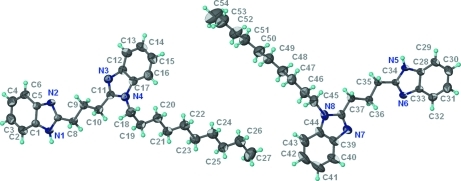
Thermal ellipsoid plot (Barbour, 2001) of two molecules of C27H26N4 at the 50% probability level; hydrogen atoms are drawn as arbitrary radius.

## supplementary crystallographic information

### Comment

Bis(2-benzimidazolyl)alkanes exhibit anti-viral activity; they are also *N*-heterocyclic compounds that form complexes with a range of metal salts. The crystal structure of the *n*-propane derivative is known as its chloride and hydrogen perchlorate salts (Hu *et al.*, 2006; Sun *et al.*, 2004). The nitrogen-bound hydrogen can be removed and the resulting anion used as a nucleophile in reacting with haloalkanes. In this study, the alkane is a ten-carbon chain; however, only one of the two H atoms can be removed. Except for the atoms of the decyl chain, the non-hydrogen atoms of the molecule of C_27_H_26_N_4_ (Scheme I) lie on an approximate plane and the –CH_2_CH_2_CH_2_– link connecting the two benzimidazolyl fused-ring systems is slightly opened up at the middle carbon (Fig. 1). The decyl substitutent adopts an extended zigzag conformation. Adjacent molecules interact by N–H···N hydrogen bonds to generate a chain parallel to the *c*-axis of the monoclinic unit cell (Table 1).

### Experimental

To 1,3-bis(1*H*-benzmidazol-2-yl)propane (0.50 g, 1.8 mmol), potassium carbonate (0.82 g, 6 mmol), and tetra-*n*-butylammonium bromide (0.01 g, 0.03 mmol) in DMF (30 ml) was added 1-bromodecane (0.75 ml, 3.6 mmol). The mixturewas stirred for 48 h. After the completion of the reaction (as monitored byTLC), the inorganic salts were filtered and the solvent was removed under reduced pressure. The residue was purified by crystallization from ethanol. Colorless crystals were isolated.

### Refinement

Carbon-bound H-atoms were placed in calculated positions (C—H 0.93–0.97 Å) and were included in the refinement in the riding model approximation, with *U*(H) set to 1.2–1.5*U*(C). The nitrogen-bound H-atoms were located in a difference Fourier map and were refined with a distance restraint of N–H 0.88±0.01 Å; their temperature factors were refined.

The decane chains of both molecules were tightly restrained by fixing the C–C distances to 1.540±0.005 Å and the 1,3-related C···C distances to 2.51±0.01 Å.

### Crystal data

**Table:**

C_27_H_36_N_4_	*F*(000) = 1808
*M**_r_* = 416.60	*D*_x_ = 1.096 Mg m^−^^3^
Monoclinic, *P*2_1_/*c*	Mo *K*α radiation, λ = 0.71073 Å
Hall symbol: -P 2ybc	Cell parameters from 8029 reflections
*a* = 38.5580 (6) Å	θ = 2.6–24.4°
*b* = 13.2052 (2) Å	µ = 0.07 mm^−^^1^
*c* = 9.9623 (2) Å	*T* = 293 K
β = 95.339 (1)°	Block, colorless
*V* = 5050.46 (15) Å^3^	0.30 × 0.20 × 0.10 mm
*Z* = 8	

### Data collection

**Table:**

Bruker APEXII diffractometer	5547 reflections with *I* > 2σ(*I*)
Radiation source: fine-focus sealed tube	*R*_int_ = 0.038
graphite	θ_max_ = 25.2°, θ_min_ = 1.6°
φ and ω scans	*h* = −46→44
38663 measured reflections	*k* = −15→14
9083 independent reflections	*l* = −11→11

### Refinement

**Table:**

Refinement on *F*^2^	Primary atom site location: structure-invariant direct methods
Least-squares matrix: full	Secondary atom site location: difference Fourier map
*R*[*F*^2^ > 2σ(*F*^2^)] = 0.056	Hydrogen site location: inferred from neighbouring sites
*wR*(*F*^2^) = 0.165	H atoms treated by a mixture of independent and constrained refinement
*S* = 1.02	*w* = 1/[σ^2^(*F*_o_^2^) + (0.0741*P*)^2^ + 1.3151*P*] where *P* = (*F*_o_^2^ + 2*F*_c_^2^)/3
9083 reflections	(Δ/σ)_max_ = 0.001
567 parameters	Δρ_max_ = 0.27 e Å^−^^3^
36 restraints	Δρ_min_ = −0.20 e Å^−^^3^

### Fractional atomic coordinates and isotropic or equivalent isotropic displacement parameters (Å^2^)

**Table:**

	*x*	*y*	*z*	*U*_iso_*/*U*_eq_	
N1	0.38712 (5)	0.73128 (16)	0.89834 (17)	0.0511 (5)	
N2	0.38956 (5)	0.73064 (15)	0.67692 (16)	0.0503 (5)	
N3	0.50737 (5)	0.84168 (15)	0.49188 (19)	0.0538 (5)	
N4	0.54455 (5)	0.94076 (14)	0.61882 (18)	0.0528 (5)	
N5	1.09927 (5)	0.23121 (15)	0.68081 (16)	0.0486 (5)	
N6	1.09643 (4)	0.21660 (15)	0.90136 (16)	0.0483 (5)	
N7	0.98714 (5)	0.37234 (17)	1.1126 (2)	0.0629 (6)	
N8	0.94086 (5)	0.42176 (16)	0.9762 (2)	0.0600 (5)	
C1	0.35798 (6)	0.67934 (18)	0.8460 (2)	0.0491 (6)	
C2	0.33103 (7)	0.6332 (2)	0.9050 (3)	0.0681 (7)	
H2	0.3301	0.6333	0.9979	0.082*	
C3	0.30579 (7)	0.5874 (2)	0.8205 (3)	0.0790 (8)	
H3	0.2873	0.5557	0.8567	0.095*	
C4	0.30719 (7)	0.5872 (2)	0.6820 (3)	0.0769 (8)	
H4	0.2895	0.5555	0.6277	0.092*	
C5	0.33388 (7)	0.6324 (2)	0.6228 (2)	0.0664 (7)	
H5A	0.3346	0.6317	0.5297	0.080*	
C6	0.35983 (6)	0.67936 (18)	0.7071 (2)	0.0497 (6)	
C7	0.40487 (5)	0.76069 (17)	0.79393 (19)	0.0448 (5)	
C8	0.43760 (6)	0.8199 (2)	0.8138 (2)	0.0550 (6)	
H8A	0.4521	0.7911	0.8889	0.066*	
H8B	0.4320	0.8888	0.8381	0.066*	
C9	0.45816 (6)	0.82310 (19)	0.6925 (2)	0.0521 (6)	
H9A	0.4435	0.8486	0.6155	0.062*	
H9B	0.4654	0.7550	0.6713	0.062*	
C10	0.48999 (6)	0.89011 (19)	0.7176 (2)	0.0551 (6)	
H10A	0.4824	0.9591	0.7310	0.066*	
H10B	0.5032	0.8684	0.8002	0.066*	
C11	0.51341 (6)	0.88951 (17)	0.6072 (2)	0.0490 (6)	
C12	0.53629 (6)	0.86276 (18)	0.4222 (2)	0.0523 (6)	
C13	0.54351 (7)	0.8351 (2)	0.2934 (3)	0.0665 (7)	
H13	0.5280	0.7953	0.2393	0.080*	
C14	0.57401 (8)	0.8679 (2)	0.2478 (3)	0.0753 (8)	
H14	0.5792	0.8502	0.1616	0.090*	
C15	0.59736 (8)	0.9274 (2)	0.3283 (3)	0.0778 (8)	
H15	0.6180	0.9478	0.2950	0.093*	
C16	0.59076 (7)	0.9566 (2)	0.4554 (3)	0.0678 (7)	
H16	0.6064	0.9964	0.5090	0.081*	
C17	0.55965 (6)	0.92400 (18)	0.5005 (2)	0.0524 (6)	
C18	0.55776 (7)	1.0071 (2)	0.7284 (2)	0.0664 (7)	
H18A	0.5383	1.0432	0.7612	0.080*	
H18B	0.5731	1.0568	0.6935	0.080*	
C19	0.57748 (6)	0.9520 (2)	0.8460 (2)	0.0704 (8)	
H19A	0.5835	1.0004	0.9177	0.084*	
H19B	0.5622	0.9019	0.8803	0.084*	
C20	0.61033 (6)	0.8999 (2)	0.8114 (2)	0.0690 (7)	
H20A	0.6254	0.9492	0.7741	0.083*	
H20B	0.6044	0.8490	0.7429	0.083*	
C21	0.62994 (6)	0.8496 (2)	0.9333 (2)	0.0752 (8)	
H21A	0.6345	0.8998	1.0038	0.090*	
H21B	0.6152	0.7976	0.9670	0.090*	
C22	0.66398 (7)	0.8024 (2)	0.9037 (3)	0.0824 (9)	
H22A	0.6782	0.8535	0.8654	0.099*	
H22B	0.6594	0.7495	0.8369	0.099*	
C23	0.68426 (7)	0.7575 (3)	1.0278 (3)	0.0852 (9)	
H23A	0.6874	0.8093	1.0968	0.102*	
H23B	0.6706	0.7034	1.0625	0.102*	
C24	0.71935 (7)	0.7162 (3)	1.0024 (3)	0.0941 (10)	
H24A	0.7327	0.7695	0.9643	0.113*	
H24B	0.7162	0.6624	0.9361	0.113*	
C25	0.74007 (7)	0.6753 (2)	1.1267 (3)	0.0823 (9)	
H25A	0.7454	0.7305	1.1896	0.099*	
H25B	0.7259	0.6266	1.1700	0.099*	
C26	0.77357 (8)	0.6252 (3)	1.0967 (3)	0.1020 (11)	
H26A	0.7683	0.5727	1.0297	0.122*	
H26B	0.7883	0.6750	1.0582	0.122*	
C27	0.79343 (10)	0.5788 (4)	1.2187 (4)	0.1507 (18)	
H27A	0.8145	0.5488	1.1932	0.226*	
H27B	0.7794	0.5276	1.2557	0.226*	
H27C	0.7990	0.6303	1.2852	0.226*	
C28	1.13001 (6)	0.18545 (18)	0.7296 (2)	0.0460 (5)	
C29	1.15871 (6)	0.1521 (2)	0.6683 (2)	0.0610 (7)	
H29	1.1597	0.1574	0.5757	0.073*	
C30	1.18557 (7)	0.1109 (2)	0.7504 (3)	0.0689 (7)	
H30	1.2054	0.0888	0.7126	0.083*	
C31	1.18397 (7)	0.1015 (2)	0.8879 (3)	0.0678 (7)	
H31	1.2027	0.0731	0.9403	0.081*	
C32	1.15530 (6)	0.1332 (2)	0.9488 (2)	0.0598 (7)	
H32	1.1543	0.1259	1.0412	0.072*	
C33	1.12790 (6)	0.17644 (17)	0.8678 (2)	0.0462 (5)	
C34	1.08043 (5)	0.24844 (17)	0.78655 (19)	0.0441 (5)	
C35	1.04563 (5)	0.29683 (19)	0.7679 (2)	0.0506 (6)	
H35A	1.0303	0.2540	0.7097	0.061*	
H35B	1.0478	0.3609	0.7218	0.061*	
C36	1.02870 (5)	0.31611 (19)	0.8968 (2)	0.0500 (6)	
H36A	1.0432	0.3617	0.9542	0.060*	
H36B	1.0267	0.2528	0.9449	0.060*	
C37	0.99280 (6)	0.3623 (2)	0.8659 (2)	0.0550 (6)	
H37A	0.9951	0.4248	0.8163	0.066*	
H37B	0.9787	0.3163	0.8079	0.066*	
C38	0.97423 (6)	0.38446 (18)	0.9873 (2)	0.0535 (6)	
C39	0.96069 (7)	0.4026 (2)	1.1895 (3)	0.0648 (7)	
C40	0.96023 (9)	0.4079 (2)	1.3284 (3)	0.0868 (9)	
H40	0.9795	0.3885	1.3857	0.104*	
C41	0.93049 (12)	0.4425 (3)	1.3787 (4)	0.1066 (12)	
H41	0.9297	0.4467	1.4715	0.128*	
C42	0.90195 (11)	0.4713 (3)	1.2950 (5)	0.1069 (13)	
H42	0.8822	0.4938	1.3329	0.128*	
C43	0.90158 (8)	0.4679 (2)	1.1571 (4)	0.0885 (10)	
H43	0.8822	0.4877	1.1008	0.106*	
C44	0.93184 (7)	0.4333 (2)	1.1062 (3)	0.0656 (7)	
C45	0.91862 (6)	0.4418 (2)	0.8529 (3)	0.0735 (8)	
H45A	0.9329	0.4643	0.7835	0.088*	
H45B	0.9026	0.4961	0.8692	0.088*	
C46	0.89794 (7)	0.3488 (2)	0.8028 (3)	0.0780 (8)	
H46A	0.9139	0.2981	0.7743	0.094*	
H46B	0.8864	0.3206	0.8766	0.094*	
C47	0.87103 (7)	0.3721 (2)	0.6869 (3)	0.0827 (9)	
H47A	0.8826	0.4005	0.6132	0.099*	
H47B	0.8551	0.4227	0.7155	0.099*	
C48	0.85046 (8)	0.2799 (2)	0.6364 (3)	0.0888 (9)	
H48A	0.8404	0.2487	0.7120	0.107*	
H48B	0.8664	0.2313	0.6025	0.107*	
C49	0.82154 (7)	0.3012 (2)	0.5267 (3)	0.0903 (10)	
H49A	0.8315	0.3289	0.4488	0.108*	
H49B	0.8060	0.3518	0.5586	0.108*	
C50	0.80075 (8)	0.2073 (3)	0.4847 (3)	0.0972 (10)	
H50A	0.8164	0.1583	0.4501	0.117*	
H50B	0.7920	0.1781	0.5644	0.117*	
C51	0.77045 (8)	0.2230 (3)	0.3803 (4)	0.1066 (11)	
H51A	0.7789	0.2512	0.2994	0.128*	
H51B	0.7545	0.2715	0.4140	0.128*	
C52	0.75103 (9)	0.1247 (3)	0.3449 (4)	0.1121 (12)	
H52A	0.7676	0.0765	0.3145	0.135*	
H52B	0.7430	0.0977	0.4270	0.135*	
C53	0.72123 (10)	0.1289 (3)	0.2430 (4)	0.1321 (15)	
H53A	0.7292	0.1519	0.1586	0.159*	
H53B	0.7047	0.1784	0.2707	0.159*	
C54	0.70289 (14)	0.0292 (3)	0.2197 (5)	0.166 (2)	
H54A	0.6842	0.0366	0.1495	0.249*	
H54B	0.6936	0.0078	0.3012	0.249*	
H54C	0.7191	−0.0206	0.1934	0.249*	
H1	0.3926 (6)	0.7455 (18)	0.9840 (12)	0.067 (8)*	
H5	1.0936 (5)	0.2486 (16)	0.5967 (12)	0.054 (7)*	

### Atomic displacement parameters (Å^2^)

**Table:**

	*U*^11^	*U*^22^	*U*^33^	*U*^12^	*U*^13^	*U*^23^
N1	0.0542 (11)	0.0698 (14)	0.0293 (10)	0.0004 (10)	0.0035 (8)	0.0004 (9)
N2	0.0523 (11)	0.0649 (13)	0.0333 (9)	−0.0004 (10)	0.0013 (8)	0.0037 (9)
N3	0.0562 (12)	0.0523 (13)	0.0528 (11)	−0.0068 (10)	0.0040 (9)	−0.0014 (9)
N4	0.0550 (11)	0.0495 (12)	0.0531 (11)	−0.0102 (10)	0.0019 (9)	0.0008 (9)
N5	0.0501 (11)	0.0691 (14)	0.0272 (9)	−0.0001 (10)	0.0067 (8)	0.0010 (9)
N6	0.0466 (10)	0.0670 (13)	0.0318 (9)	0.0005 (10)	0.0063 (8)	−0.0005 (8)
N7	0.0643 (13)	0.0711 (15)	0.0555 (12)	0.0132 (12)	0.0164 (10)	0.0023 (10)
N8	0.0473 (11)	0.0574 (14)	0.0766 (14)	0.0082 (10)	0.0122 (10)	−0.0005 (11)
C1	0.0493 (13)	0.0556 (15)	0.0426 (12)	0.0046 (12)	0.0055 (10)	0.0024 (11)
C2	0.0650 (16)	0.081 (2)	0.0611 (15)	−0.0060 (15)	0.0194 (13)	0.0051 (14)
C3	0.0662 (17)	0.083 (2)	0.090 (2)	−0.0165 (16)	0.0195 (16)	−0.0003 (17)
C4	0.0670 (17)	0.079 (2)	0.083 (2)	−0.0123 (16)	−0.0029 (15)	−0.0106 (16)
C5	0.0666 (16)	0.079 (2)	0.0520 (14)	−0.0061 (15)	−0.0041 (13)	−0.0058 (13)
C6	0.0503 (13)	0.0577 (15)	0.0404 (12)	0.0042 (12)	0.0001 (10)	0.0012 (11)
C7	0.0485 (12)	0.0542 (15)	0.0311 (11)	0.0058 (11)	0.0010 (9)	0.0017 (10)
C8	0.0553 (14)	0.0682 (17)	0.0407 (12)	−0.0033 (13)	0.0008 (10)	−0.0001 (11)
C9	0.0501 (13)	0.0611 (16)	0.0450 (12)	0.0012 (12)	0.0042 (10)	0.0031 (11)
C10	0.0570 (14)	0.0544 (15)	0.0536 (13)	−0.0014 (13)	0.0030 (11)	−0.0033 (11)
C11	0.0517 (13)	0.0452 (14)	0.0494 (13)	−0.0004 (12)	0.0017 (10)	0.0036 (11)
C12	0.0547 (14)	0.0466 (15)	0.0556 (14)	−0.0005 (12)	0.0046 (11)	0.0041 (11)
C13	0.0796 (18)	0.0598 (17)	0.0614 (16)	−0.0045 (15)	0.0140 (14)	−0.0022 (13)
C14	0.091 (2)	0.073 (2)	0.0658 (17)	0.0028 (18)	0.0291 (16)	0.0024 (15)
C15	0.0711 (18)	0.080 (2)	0.087 (2)	−0.0048 (17)	0.0299 (16)	0.0125 (17)
C16	0.0627 (16)	0.0676 (19)	0.0736 (18)	−0.0124 (14)	0.0084 (14)	0.0065 (14)
C17	0.0549 (14)	0.0462 (15)	0.0562 (14)	−0.0035 (12)	0.0057 (11)	0.0067 (11)
C18	0.0691 (16)	0.0594 (17)	0.0702 (17)	−0.0147 (14)	0.0035 (13)	−0.0130 (13)
C19	0.0655 (16)	0.087 (2)	0.0580 (15)	−0.0154 (16)	0.0017 (13)	−0.0142 (14)
C20	0.0679 (17)	0.082 (2)	0.0571 (15)	−0.0145 (15)	0.0031 (13)	0.0005 (14)
C21	0.0692 (17)	0.099 (2)	0.0578 (15)	−0.0091 (17)	0.0063 (13)	−0.0021 (15)
C22	0.0798 (19)	0.105 (2)	0.0627 (17)	−0.0010 (18)	0.0102 (14)	0.0103 (16)
C23	0.081 (2)	0.107 (3)	0.0690 (18)	0.0008 (19)	0.0129 (15)	0.0071 (17)
C24	0.090 (2)	0.124 (3)	0.0691 (19)	0.006 (2)	0.0107 (16)	0.0025 (18)
C25	0.0752 (19)	0.101 (2)	0.0703 (18)	−0.0099 (18)	0.0050 (15)	−0.0006 (17)
C26	0.086 (2)	0.122 (3)	0.100 (2)	−0.008 (2)	0.0190 (19)	−0.021 (2)
C27	0.110 (3)	0.187 (5)	0.146 (4)	0.032 (3)	−0.040 (3)	−0.016 (3)
C28	0.0467 (12)	0.0526 (15)	0.0396 (11)	−0.0016 (11)	0.0083 (10)	−0.0027 (10)
C29	0.0625 (15)	0.0705 (18)	0.0528 (14)	0.0052 (14)	0.0207 (12)	−0.0015 (12)
C30	0.0580 (15)	0.0743 (19)	0.0768 (18)	0.0144 (14)	0.0186 (14)	−0.0008 (15)
C31	0.0568 (15)	0.0716 (19)	0.0741 (18)	0.0103 (14)	0.0008 (13)	0.0056 (14)
C32	0.0604 (15)	0.0720 (18)	0.0463 (13)	0.0008 (14)	0.0010 (11)	0.0055 (12)
C33	0.0472 (13)	0.0544 (15)	0.0373 (11)	−0.0029 (11)	0.0046 (10)	−0.0006 (10)
C34	0.0445 (12)	0.0563 (15)	0.0319 (11)	−0.0048 (11)	0.0062 (9)	−0.0041 (10)
C35	0.0454 (12)	0.0688 (16)	0.0380 (11)	0.0002 (12)	0.0050 (9)	−0.0014 (11)
C36	0.0448 (12)	0.0637 (16)	0.0420 (12)	−0.0001 (12)	0.0061 (9)	−0.0059 (11)
C37	0.0493 (13)	0.0651 (17)	0.0509 (13)	0.0039 (12)	0.0066 (10)	−0.0015 (12)
C38	0.0479 (13)	0.0522 (15)	0.0622 (15)	0.0034 (12)	0.0139 (11)	−0.0004 (12)
C39	0.0733 (17)	0.0580 (17)	0.0678 (16)	0.0045 (14)	0.0313 (14)	0.0014 (13)
C40	0.111 (2)	0.081 (2)	0.0744 (19)	0.0117 (19)	0.0398 (18)	0.0036 (16)
C41	0.136 (3)	0.099 (3)	0.097 (3)	0.000 (3)	0.070 (3)	−0.006 (2)
C42	0.105 (3)	0.092 (3)	0.136 (3)	0.002 (2)	0.077 (3)	−0.022 (2)
C43	0.0702 (19)	0.073 (2)	0.128 (3)	0.0043 (16)	0.0427 (19)	−0.0122 (19)
C44	0.0608 (16)	0.0524 (17)	0.0884 (19)	0.0015 (14)	0.0316 (15)	−0.0038 (14)
C45	0.0552 (15)	0.070 (2)	0.094 (2)	0.0150 (15)	0.0035 (14)	0.0084 (16)
C46	0.0695 (17)	0.079 (2)	0.0837 (19)	0.0062 (17)	−0.0018 (15)	0.0002 (16)
C47	0.0639 (17)	0.102 (3)	0.082 (2)	0.0096 (18)	0.0072 (15)	0.0118 (18)
C48	0.089 (2)	0.094 (2)	0.081 (2)	0.007 (2)	−0.0025 (17)	−0.0081 (18)
C49	0.081 (2)	0.108 (3)	0.080 (2)	0.009 (2)	−0.0016 (16)	−0.0008 (18)
C50	0.098 (2)	0.104 (3)	0.086 (2)	0.006 (2)	−0.0047 (18)	−0.0102 (19)
C51	0.088 (2)	0.127 (3)	0.102 (3)	0.009 (2)	−0.0071 (19)	−0.010 (2)
C52	0.128 (3)	0.104 (3)	0.100 (3)	0.002 (3)	−0.014 (2)	0.000 (2)
C53	0.136 (3)	0.138 (4)	0.116 (3)	−0.010 (3)	−0.026 (3)	0.028 (3)
C54	0.224 (5)	0.113 (4)	0.146 (4)	−0.050 (4)	−0.062 (4)	0.030 (3)

### Geometric parameters (Å, °)

**Table:**

N1—C7	1.354 (3)	C24—H24B	0.9700
N1—C1	1.377 (3)	C25—C26	1.507 (3)
N1—H1	0.880 (10)	C25—H25A	0.9700
N2—C7	1.318 (3)	C25—H25B	0.9700
N2—C6	1.388 (3)	C26—C27	1.505 (4)
N3—C11	1.313 (3)	C26—H26A	0.9700
N3—C12	1.395 (3)	C26—H26B	0.9700
N4—C11	1.374 (3)	C27—H27A	0.9600
N4—C17	1.381 (3)	C27—H27B	0.9600
N4—C18	1.454 (3)	C27—H27C	0.9600
N5—C34	1.353 (3)	C28—C29	1.384 (3)
N5—C28	1.378 (3)	C28—C33	1.392 (3)
N5—H5	0.876 (10)	C29—C30	1.371 (3)
N6—C34	1.317 (3)	C29—H29	0.9300
N6—C33	1.393 (3)	C30—C31	1.383 (3)
N7—C38	1.310 (3)	C30—H30	0.9300
N7—C39	1.390 (3)	C31—C32	1.375 (3)
N8—C38	1.372 (3)	C31—H31	0.9300
N8—C44	1.381 (3)	C32—C33	1.391 (3)
N8—C45	1.455 (3)	C32—H32	0.9300
C1—C2	1.382 (3)	C34—C35	1.482 (3)
C1—C6	1.393 (3)	C35—C36	1.515 (3)
C2—C3	1.367 (4)	C35—H35A	0.9700
C2—H2	0.9300	C35—H35B	0.9700
C3—C4	1.386 (4)	C36—C37	1.518 (3)
C3—H3	0.9300	C36—H36A	0.9700
C4—C5	1.370 (4)	C36—H36B	0.9700
C4—H4	0.9300	C37—C38	1.491 (3)
C5—C6	1.391 (3)	C37—H37A	0.9700
C5—H5A	0.9300	C37—H37B	0.9700
C7—C8	1.483 (3)	C39—C44	1.386 (4)
C8—C9	1.507 (3)	C39—C40	1.387 (4)
C8—H8A	0.9700	C40—C41	1.372 (4)
C8—H8B	0.9700	C40—H40	0.9300
C9—C10	1.515 (3)	C41—C42	1.371 (5)
C9—H9A	0.9700	C41—H41	0.9300
C9—H9B	0.9700	C42—C43	1.373 (5)
C10—C11	1.486 (3)	C42—H42	0.9300
C10—H10A	0.9700	C43—C44	1.392 (4)
C10—H10B	0.9700	C43—H43	0.9300
C12—C13	1.387 (3)	C45—C46	1.523 (3)
C12—C17	1.394 (3)	C45—H45A	0.9700
C13—C14	1.370 (4)	C45—H45B	0.9700
C13—H13	0.9300	C46—C47	1.510 (3)
C14—C15	1.391 (4)	C46—H46A	0.9700
C14—H14	0.9300	C46—H46B	0.9700
C15—C16	1.370 (4)	C47—C48	1.513 (3)
C15—H15	0.9300	C47—H47A	0.9700
C16—C17	1.388 (3)	C47—H47B	0.9700
C16—H16	0.9300	C48—C49	1.513 (3)
C18—C19	1.521 (3)	C48—H48A	0.9700
C18—H18A	0.9700	C48—H48B	0.9700
C18—H18B	0.9700	C49—C50	1.514 (3)
C19—C20	1.509 (3)	C49—H49A	0.9700
C19—H19A	0.9700	C49—H49B	0.9700
C19—H19B	0.9700	C50—C51	1.504 (3)
C20—C21	1.522 (3)	C50—H50A	0.9700
C20—H20A	0.9700	C50—H50B	0.9700
C20—H20B	0.9700	C51—C52	1.525 (4)
C21—C22	1.507 (3)	C51—H51A	0.9700
C21—H21A	0.9700	C51—H51B	0.9700
C21—H21B	0.9700	C52—C53	1.462 (3)
C22—C23	1.521 (3)	C52—H52A	0.9700
C22—H22A	0.9700	C52—H52B	0.9700
C22—H22B	0.9700	C53—C54	1.502 (4)
C23—C24	1.502 (3)	C53—H53A	0.9700
C23—H23A	0.9700	C53—H53B	0.9700
C23—H23B	0.9700	C54—H54A	0.9600
C24—C25	1.509 (3)	C54—H54B	0.9600
C24—H24A	0.9700	C54—H54C	0.9600
			
C7—N1—C1	107.76 (17)	C25—C26—H26B	108.9
C7—N1—H1	126.1 (16)	H26A—C26—H26B	107.7
C1—N1—H1	126.0 (16)	C26—C27—H27A	109.5
C7—N2—C6	105.45 (17)	C26—C27—H27B	109.5
C11—N3—C12	104.76 (19)	H27A—C27—H27B	109.5
C11—N4—C17	106.49 (18)	C26—C27—H27C	109.5
C11—N4—C18	127.0 (2)	H27A—C27—H27C	109.5
C17—N4—C18	126.3 (2)	H27B—C27—H27C	109.5
C34—N5—C28	107.81 (17)	N5—C28—C29	132.9 (2)
C34—N5—H5	126.6 (14)	N5—C28—C33	104.99 (18)
C28—N5—H5	125.5 (14)	C29—C28—C33	122.1 (2)
C34—N6—C33	105.14 (16)	C30—C29—C28	117.0 (2)
C38—N7—C39	104.9 (2)	C30—C29—H29	121.5
C38—N8—C44	106.3 (2)	C28—C29—H29	121.5
C38—N8—C45	127.4 (2)	C29—C30—C31	121.7 (2)
C44—N8—C45	126.3 (2)	C29—C30—H30	119.1
N1—C1—C2	132.7 (2)	C31—C30—H30	119.1
N1—C1—C6	105.10 (19)	C32—C31—C30	121.5 (2)
C2—C1—C6	122.2 (2)	C32—C31—H31	119.3
C3—C2—C1	117.0 (2)	C30—C31—H31	119.3
C3—C2—H2	121.5	C31—C32—C33	117.8 (2)
C1—C2—H2	121.5	C31—C32—H32	121.1
C2—C3—C4	121.5 (3)	C33—C32—H32	121.1
C2—C3—H3	119.2	C32—C33—C28	119.9 (2)
C4—C3—H3	119.2	C32—C33—N6	130.44 (19)
C5—C4—C3	121.9 (3)	C28—C33—N6	109.70 (18)
C5—C4—H4	119.1	N6—C34—N5	112.36 (19)
C3—C4—H4	119.1	N6—C34—C35	126.30 (18)
C4—C5—C6	117.5 (2)	N5—C34—C35	121.34 (18)
C4—C5—H5A	121.3	C34—C35—C36	114.99 (18)
C6—C5—H5A	121.3	C34—C35—H35A	108.5
N2—C6—C5	130.5 (2)	C36—C35—H35A	108.5
N2—C6—C1	109.56 (19)	C34—C35—H35B	108.5
C5—C6—C1	120.0 (2)	C36—C35—H35B	108.5
N2—C7—N1	112.1 (2)	H35A—C35—H35B	107.5
N2—C7—C8	125.65 (19)	C35—C36—C37	110.62 (17)
N1—C7—C8	122.23 (18)	C35—C36—H36A	109.5
C7—C8—C9	114.34 (18)	C37—C36—H36A	109.5
C7—C8—H8A	108.7	C35—C36—H36B	109.5
C9—C8—H8A	108.7	C37—C36—H36B	109.5
C7—C8—H8B	108.7	H36A—C36—H36B	108.1
C9—C8—H8B	108.7	C38—C37—C36	114.32 (19)
H8A—C8—H8B	107.6	C38—C37—H37A	108.7
C8—C9—C10	111.25 (19)	C36—C37—H37A	108.7
C8—C9—H9A	109.4	C38—C37—H37B	108.7
C10—C9—H9A	109.4	C36—C37—H37B	108.7
C8—C9—H9B	109.4	H37A—C37—H37B	107.6
C10—C9—H9B	109.4	N7—C38—N8	113.0 (2)
H9A—C9—H9B	108.0	N7—C38—C37	125.5 (2)
C11—C10—C9	114.42 (19)	N8—C38—C37	121.5 (2)
C11—C10—H10A	108.7	C44—C39—C40	119.9 (3)
C9—C10—H10A	108.7	C44—C39—N7	110.1 (2)
C11—C10—H10B	108.7	C40—C39—N7	130.0 (3)
C9—C10—H10B	108.7	C41—C40—C39	118.0 (3)
H10A—C10—H10B	107.6	C41—C40—H40	121.0
N3—C11—N4	113.15 (19)	C39—C40—H40	121.0
N3—C11—C10	125.4 (2)	C42—C41—C40	121.4 (3)
N4—C11—C10	121.4 (2)	C42—C41—H41	119.3
C13—C12—C17	119.7 (2)	C40—C41—H41	119.3
C13—C12—N3	130.3 (2)	C41—C42—C43	122.2 (3)
C17—C12—N3	110.0 (2)	C41—C42—H42	118.9
C14—C13—C12	118.4 (3)	C43—C42—H42	118.9
C14—C13—H13	120.8	C42—C43—C44	116.4 (3)
C12—C13—H13	120.8	C42—C43—H43	121.8
C13—C14—C15	121.1 (3)	C44—C43—H43	121.8
C13—C14—H14	119.4	N8—C44—C39	105.7 (2)
C15—C14—H14	119.4	N8—C44—C43	132.2 (3)
C16—C15—C14	121.7 (3)	C39—C44—C43	122.1 (3)
C16—C15—H15	119.1	N8—C45—C46	112.3 (2)
C14—C15—H15	119.1	N8—C45—H45A	109.1
C15—C16—C17	116.9 (3)	C46—C45—H45A	109.1
C15—C16—H16	121.6	N8—C45—H45B	109.1
C17—C16—H16	121.6	C46—C45—H45B	109.1
N4—C17—C16	132.3 (2)	H45A—C45—H45B	107.9
N4—C17—C12	105.60 (19)	C47—C46—C45	112.9 (2)
C16—C17—C12	122.1 (2)	C47—C46—H46A	109.0
N4—C18—C19	113.9 (2)	C45—C46—H46A	109.0
N4—C18—H18A	108.8	C47—C46—H46B	109.0
C19—C18—H18A	108.8	C45—C46—H46B	109.0
N4—C18—H18B	108.8	H46A—C46—H46B	107.8
C19—C18—H18B	108.8	C46—C47—C48	113.1 (2)
H18A—C18—H18B	107.7	C46—C47—H47A	109.0
C20—C19—C18	114.3 (2)	C48—C47—H47A	109.0
C20—C19—H19A	108.7	C46—C47—H47B	109.0
C18—C19—H19A	108.7	C48—C47—H47B	109.0
C20—C19—H19B	108.7	H47A—C47—H47B	107.8
C18—C19—H19B	108.7	C47—C48—C49	114.7 (3)
H19A—C19—H19B	107.6	C47—C48—H48A	108.6
C19—C20—C21	112.5 (2)	C49—C48—H48A	108.6
C19—C20—H20A	109.1	C47—C48—H48B	108.6
C21—C20—H20A	109.1	C49—C48—H48B	108.6
C19—C20—H20B	109.1	H48A—C48—H48B	107.6
C21—C20—H20B	109.1	C48—C49—C50	112.5 (3)
H20A—C20—H20B	107.8	C48—C49—H49A	109.1
C22—C21—C20	113.7 (2)	C50—C49—H49A	109.1
C22—C21—H21A	108.8	C48—C49—H49B	109.1
C20—C21—H21A	108.8	C50—C49—H49B	109.1
C22—C21—H21B	108.8	H49A—C49—H49B	107.8
C20—C21—H21B	108.8	C51—C50—C49	115.8 (3)
H21A—C21—H21B	107.7	C51—C50—H50A	108.3
C21—C22—C23	113.2 (2)	C49—C50—H50A	108.3
C21—C22—H22A	108.9	C51—C50—H50B	108.3
C23—C22—H22A	108.9	C49—C50—H50B	108.3
C21—C22—H22B	108.9	H50A—C50—H50B	107.4
C23—C22—H22B	108.9	C50—C51—C52	112.1 (3)
H22A—C22—H22B	107.8	C50—C51—H51A	109.2
C24—C23—C22	114.1 (2)	C52—C51—H51A	109.2
C24—C23—H23A	108.7	C50—C51—H51B	109.2
C22—C23—H23A	108.7	C52—C51—H51B	109.2
C24—C23—H23B	108.7	H51A—C51—H51B	107.9
C22—C23—H23B	108.7	C53—C52—C51	117.8 (3)
H23A—C23—H23B	107.6	C53—C52—H52A	107.9
C23—C24—C25	114.1 (2)	C51—C52—H52A	107.9
C23—C24—H24A	108.7	C53—C52—H52B	107.9
C25—C24—H24A	108.7	C51—C52—H52B	107.9
C23—C24—H24B	108.7	H52A—C52—H52B	107.2
C25—C24—H24B	108.7	C52—C53—C54	113.7 (3)
H24A—C24—H24B	107.6	C52—C53—H53A	108.8
C26—C25—C24	113.1 (2)	C54—C53—H53A	108.8
C26—C25—H25A	109.0	C52—C53—H53B	108.8
C24—C25—H25A	109.0	C54—C53—H53B	108.8
C26—C25—H25B	109.0	H53A—C53—H53B	107.7
C24—C25—H25B	109.0	C53—C54—H54A	109.5
H25A—C25—H25B	107.8	C53—C54—H54B	109.5
C27—C26—C25	113.5 (3)	H54A—C54—H54B	109.5
C27—C26—H26A	108.9	C53—C54—H54C	109.5
C25—C26—H26A	108.9	H54A—C54—H54C	109.5
C27—C26—H26B	108.9	H54B—C54—H54C	109.5
			
C7—N1—C1—C2	−179.9 (3)	C34—N5—C28—C29	−179.4 (3)
C7—N1—C1—C6	0.5 (2)	C34—N5—C28—C33	0.4 (2)
N1—C1—C2—C3	−179.9 (3)	N5—C28—C29—C30	178.7 (2)
C6—C1—C2—C3	−0.4 (4)	C33—C28—C29—C30	−1.1 (4)
C1—C2—C3—C4	0.0 (4)	C28—C29—C30—C31	1.0 (4)
C2—C3—C4—C5	0.2 (5)	C29—C30—C31—C32	−0.1 (4)
C3—C4—C5—C6	−0.2 (4)	C30—C31—C32—C33	−0.8 (4)
C7—N2—C6—C5	179.5 (3)	C31—C32—C33—C28	0.6 (4)
C7—N2—C6—C1	−0.3 (3)	C31—C32—C33—N6	−178.5 (2)
C4—C5—C6—N2	−179.9 (2)	N5—C28—C33—C32	−179.6 (2)
C4—C5—C6—C1	−0.1 (4)	C29—C28—C33—C32	0.3 (4)
N1—C1—C6—N2	−0.1 (3)	N5—C28—C33—N6	−0.2 (3)
C2—C1—C6—N2	−179.8 (2)	C29—C28—C33—N6	179.6 (2)
N1—C1—C6—C5	−179.9 (2)	C34—N6—C33—C32	179.2 (2)
C2—C1—C6—C5	0.4 (4)	C34—N6—C33—C28	−0.1 (3)
C6—N2—C7—N1	0.6 (3)	C33—N6—C34—N5	0.3 (3)
C6—N2—C7—C8	−179.3 (2)	C33—N6—C34—C35	179.5 (2)
C1—N1—C7—N2	−0.7 (3)	C28—N5—C34—N6	−0.5 (3)
C1—N1—C7—C8	179.2 (2)	C28—N5—C34—C35	−179.7 (2)
N2—C7—C8—C9	−14.7 (3)	N6—C34—C35—C36	4.1 (3)
N1—C7—C8—C9	165.4 (2)	N5—C34—C35—C36	−176.8 (2)
C7—C8—C9—C10	176.5 (2)	C34—C35—C36—C37	−178.0 (2)
C8—C9—C10—C11	174.6 (2)	C35—C36—C37—C38	−179.7 (2)
C12—N3—C11—N4	−0.2 (3)	C39—N7—C38—N8	0.5 (3)
C12—N3—C11—C10	−179.8 (2)	C39—N7—C38—C37	179.9 (2)
C17—N4—C11—N3	0.0 (3)	C44—N8—C38—N7	−0.6 (3)
C18—N4—C11—N3	175.0 (2)	C45—N8—C38—N7	−178.4 (2)
C17—N4—C11—C10	179.6 (2)	C44—N8—C38—C37	−180.0 (2)
C18—N4—C11—C10	−5.4 (3)	C45—N8—C38—C37	2.2 (4)
C9—C10—C11—N3	4.2 (3)	C36—C37—C38—N7	3.9 (4)
C9—C10—C11—N4	−175.4 (2)	C36—C37—C38—N8	−176.8 (2)
C11—N3—C12—C13	−177.5 (3)	C38—N7—C39—C44	−0.3 (3)
C11—N3—C12—C17	0.4 (3)	C38—N7—C39—C40	−178.3 (3)
C17—C12—C13—C14	1.1 (4)	C44—C39—C40—C41	1.0 (4)
N3—C12—C13—C14	178.8 (2)	N7—C39—C40—C41	178.9 (3)
C12—C13—C14—C15	0.1 (4)	C39—C40—C41—C42	0.0 (5)
C13—C14—C15—C16	−0.8 (5)	C40—C41—C42—C43	−0.6 (6)
C14—C15—C16—C17	0.1 (4)	C41—C42—C43—C44	0.2 (5)
C11—N4—C17—C16	179.7 (3)	C38—N8—C44—C39	0.3 (3)
C18—N4—C17—C16	4.7 (4)	C45—N8—C44—C39	178.3 (2)
C11—N4—C17—C12	0.2 (2)	C38—N8—C44—C43	180.0 (3)
C18—N4—C17—C12	−174.8 (2)	C45—N8—C44—C43	−2.1 (5)
C15—C16—C17—N4	−178.2 (3)	C40—C39—C44—N8	178.2 (2)
C15—C16—C17—C12	1.2 (4)	N7—C39—C44—N8	−0.1 (3)
C13—C12—C17—N4	177.7 (2)	C40—C39—C44—C43	−1.4 (4)
N3—C12—C17—N4	−0.4 (3)	N7—C39—C44—C43	−179.7 (2)
C13—C12—C17—C16	−1.8 (4)	C42—C43—C44—N8	−178.7 (3)
N3—C12—C17—C16	−180.0 (2)	C42—C43—C44—C39	0.9 (4)
C11—N4—C18—C19	86.5 (3)	C38—N8—C45—C46	87.3 (3)
C17—N4—C18—C19	−99.4 (3)	C44—N8—C45—C46	−90.2 (3)
N4—C18—C19—C20	63.6 (3)	N8—C45—C46—C47	171.8 (2)
C18—C19—C20—C21	177.6 (2)	C45—C46—C47—C48	179.9 (3)
C19—C20—C21—C22	−176.4 (2)	C46—C47—C48—C49	176.0 (3)
C20—C21—C22—C23	176.9 (3)	C47—C48—C49—C50	−177.3 (3)
C21—C22—C23—C24	−176.1 (3)	C48—C49—C50—C51	177.3 (3)
C22—C23—C24—C25	177.7 (3)	C49—C50—C51—C52	−179.8 (3)
C23—C24—C25—C26	174.3 (3)	C50—C51—C52—C53	−179.2 (3)
C24—C25—C26—C27	−176.6 (3)	C51—C52—C53—C54	−177.4 (4)

### Hydrogen-bond geometry (Å, °)

**Table:**

*D*—H···*A*	*D*—H	H···*A*	*D*···*A*	*D*—H···*A*
N1—H1···N2^i^	0.88 (1)	1.96 (1)	2.813 (2)	162 (2)
N5—H5···N6^ii^	0.88 (1)	2.01 (1)	2.860 (2)	162 (2)

Symmetry codes: (i) *x*, −*y*+3/2, *z*+1/2; (ii) *x*, −*y*+1/2, *z*−1/2.
